# Supplementary material for: Metagenomic Sequencing Reveals that High-Grain Feeding Alters the Composition and Metabolism of Cecal Microbiota and Induces Cecal Mucosal Injury in Sheep
Source: mSystems. 2021 Oct 5;6(5):e00915-21. doi: 10.1128/mSystems.00915-21 (PMC8547435; doi:10.1128/mSystems.00915-21)
Supplement: TABLE S1 [file msystems.00915-21-st001.docx]

**Table S1. pHs and concentrations of VFAs and lactate in the cecal digesta of sheep among the CON and HG groups.**

| Items | Treatment | | | | SEM^1^ | *P* value^2^ |
| --- | --- | --- | --- | --- | --- | --- |
|  | CON | HG7 | HG14 | HG28 |  |  |
| pH | 6.85^a^ | 6.37^bc^ | 6.61^b^ | 6.28^c^ | 0.07 | 0.005 |
| Concentration, μmol/g | | | | | | |
| Total VFA^3^ | 29.27^c^ | 38.08^b^ | 43.79^b^ | 51.18^a^ | 1.98 | 0.001 |
| Acetate | 23.33^c^ | 29.64^b^ | 33.72^b^ | 39.56^a^ | 1.50 | 0.001 |
| Propionate | 4.04 | 4.10 | 4.40 | 5.17 | 0.28 | 0.658 |
| Butyrate | 0.97^c^ | 3.87^b^ | 4.96^a^ | 5.48^a^ | 0.41 | 0.001 |
| Valerate | 0.21^b^ | 0.11^c^ | 0.22^b^ | 0.32^a^ | 0.02 | 0.001 |
| Isobutyrate | 0.46^a^ | 0.18^b^ | 0.34^a^ | 0.46^a^ | 0.04 | 0.008 |
| Isovalerate | 0.26^a^ | 0.18^ab^ | 0.16^b^ | 0.18^ab^ | 0.01 | 0.029 |
| Lactate | 1.93 | 2.26 | 2.25 | 4.03 | 0.41 | 0.268 |
| Butyrate:acetate | 0.04^b^ | 0.13^a^ | 0.15^a^ | 0.14^a^ | 0.01 | 0.009 |
| Proportion, % |  |  |  |  |  |  |
| Acetate | 79.71 | 77.75 | 76.92 | 77.39 | 0.63 | 0.226 |
| Propionate | 13.82 | 10.61 | 10.08 | 10.02 | 0.65 | 0.066 |
| Butyrate | 3.32^b^ | 10.40^a^ | 11.36^a^ | 10.70^a^ | 0.81 | 0.008 |
| Valerate | 0.70^a^ | 0.30^c^ | 0.50^b^ | 0.63^ab^ | 0.11 | 0.003 |
| Isobutyrate | 1.57^a^ | 0.46^c^ | 0.77^b^ | 0.90^b^ | 0.11 | 0.020 |
| Isovalerate | 0.88^a^ | 0.48^b^ | 0.37^c^ | 0.36^c^ | 0.11 | 0.003 |

^1^SEM, standard error of the mean; ^2^*P* value, The Kruskal–Wallis rank-sum test was used to detect the significance of the four groups; ^3^VFA, volatile fatty acids. Different letters represent significant differences between the four groups (Wilcoxon rank-sum test, *P* value < 0.05).
