# Supplementary figures and images for: Metagenomic Sequencing Reveals that High-Grain Feeding Alters the Composition and Metabolism of Cecal Microbiota and Induces Cecal Mucosal Injury in Sheep
Source: mSystems. 2021 Oct 5;6(5):e00915-21. doi: 10.1128/mSystems.00915-21 (PMC8547435; doi:10.1128/mSystems.00915-21)

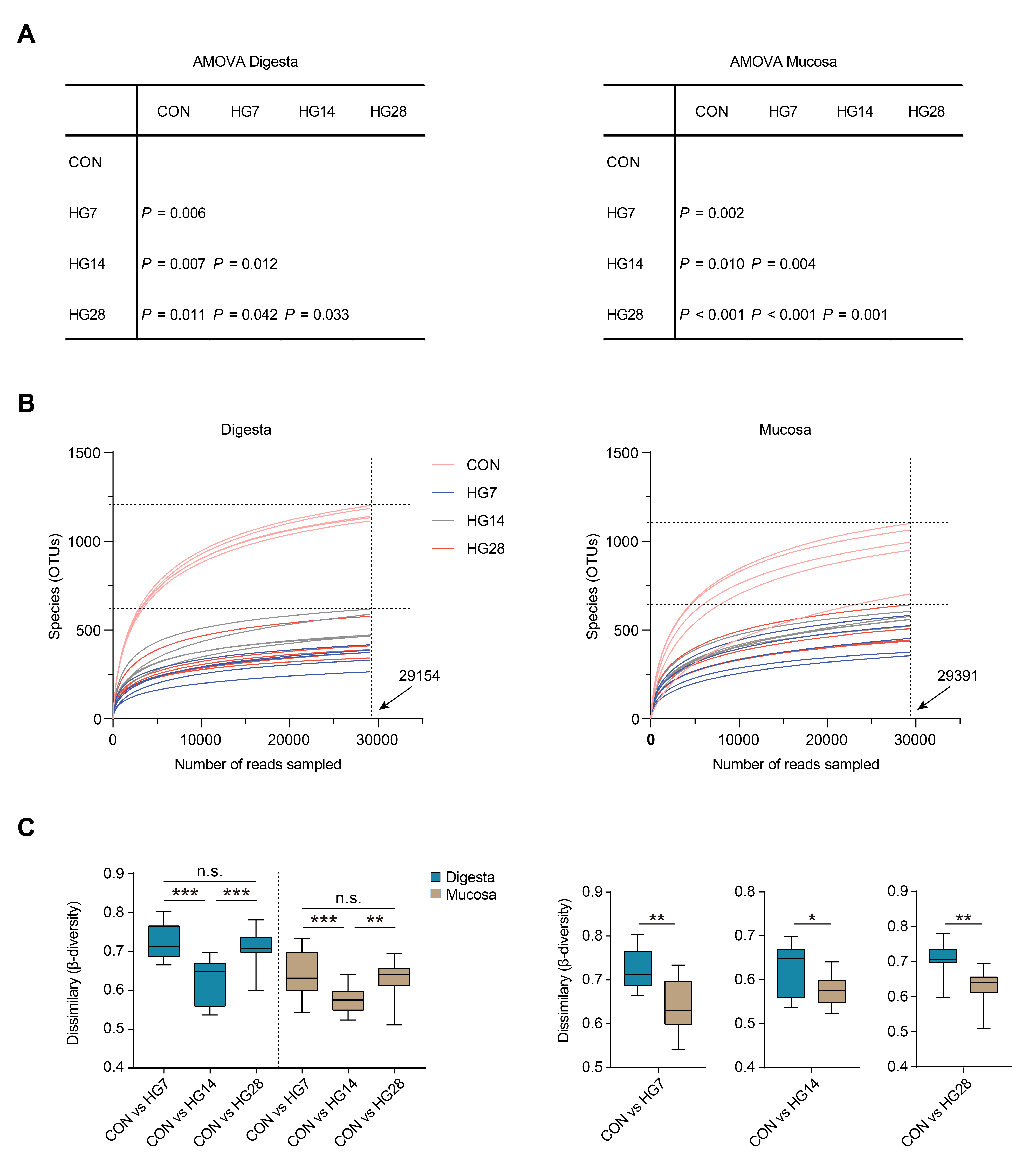

Supplement: FIG S1 [file msystems.00915-21-sf001.tif]

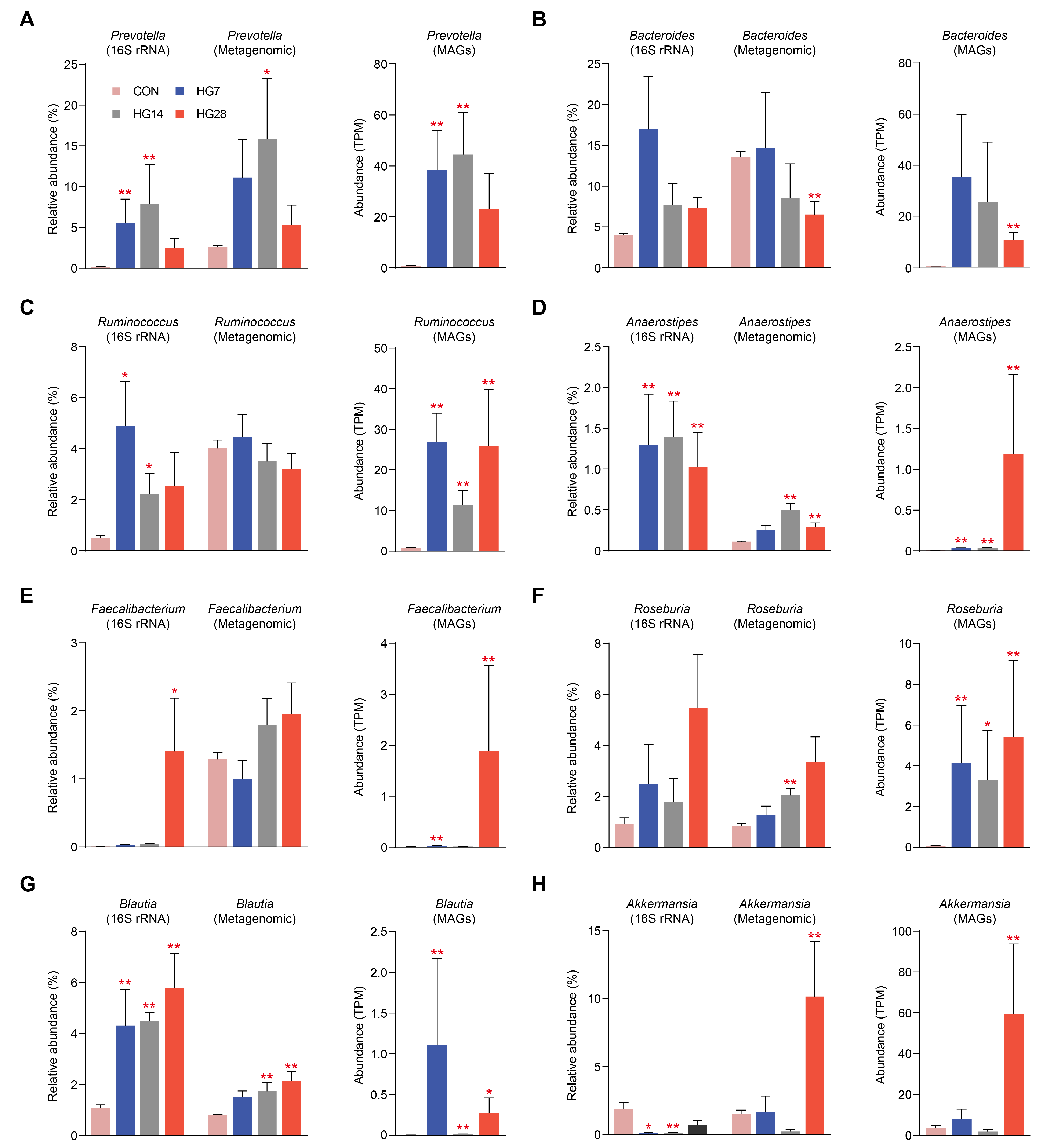

Supplement: FIG S2 [file msystems.00915-21-sf002.tif]

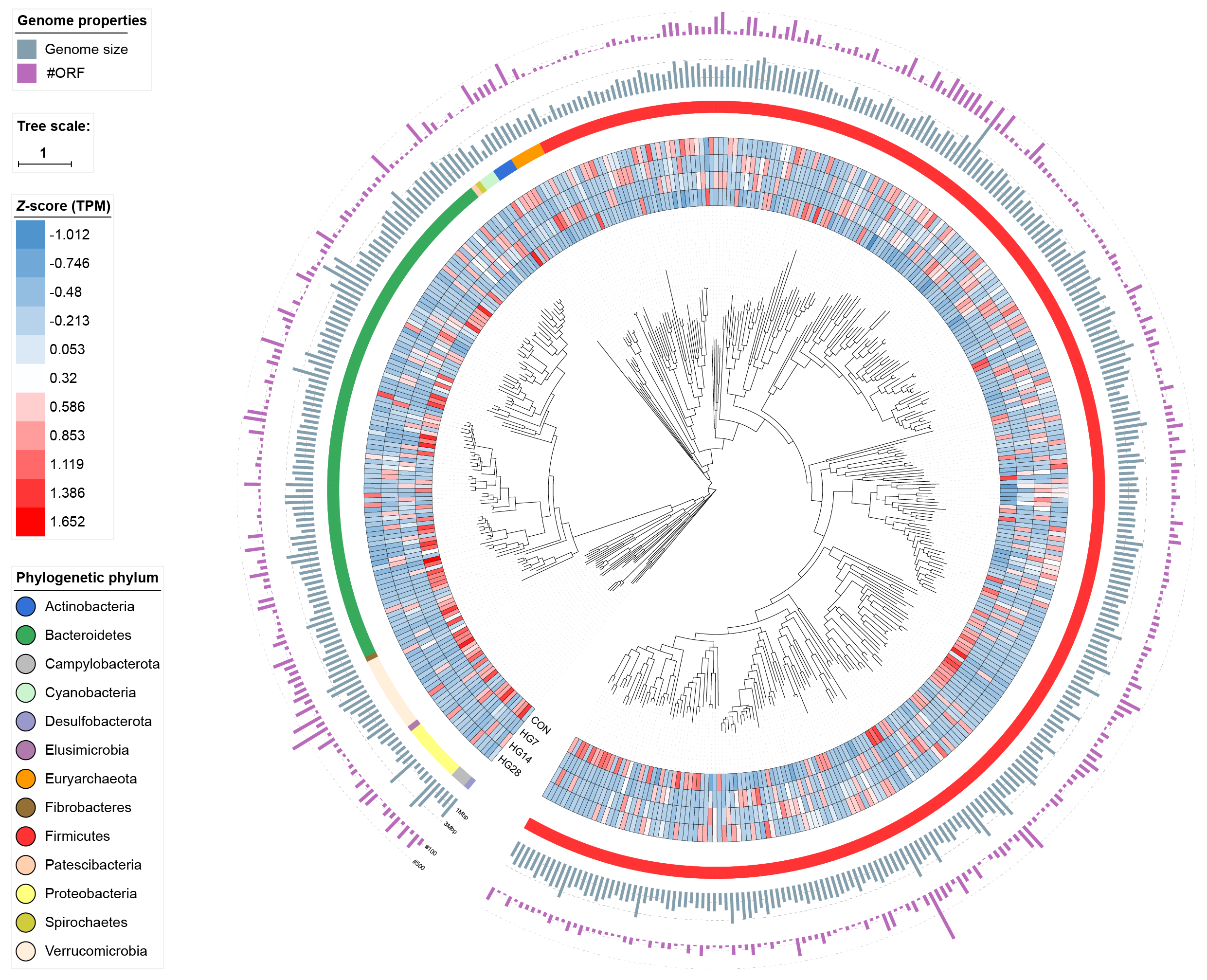

Supplement: FIG S3 [file msystems.00915-21-sf003.tif]

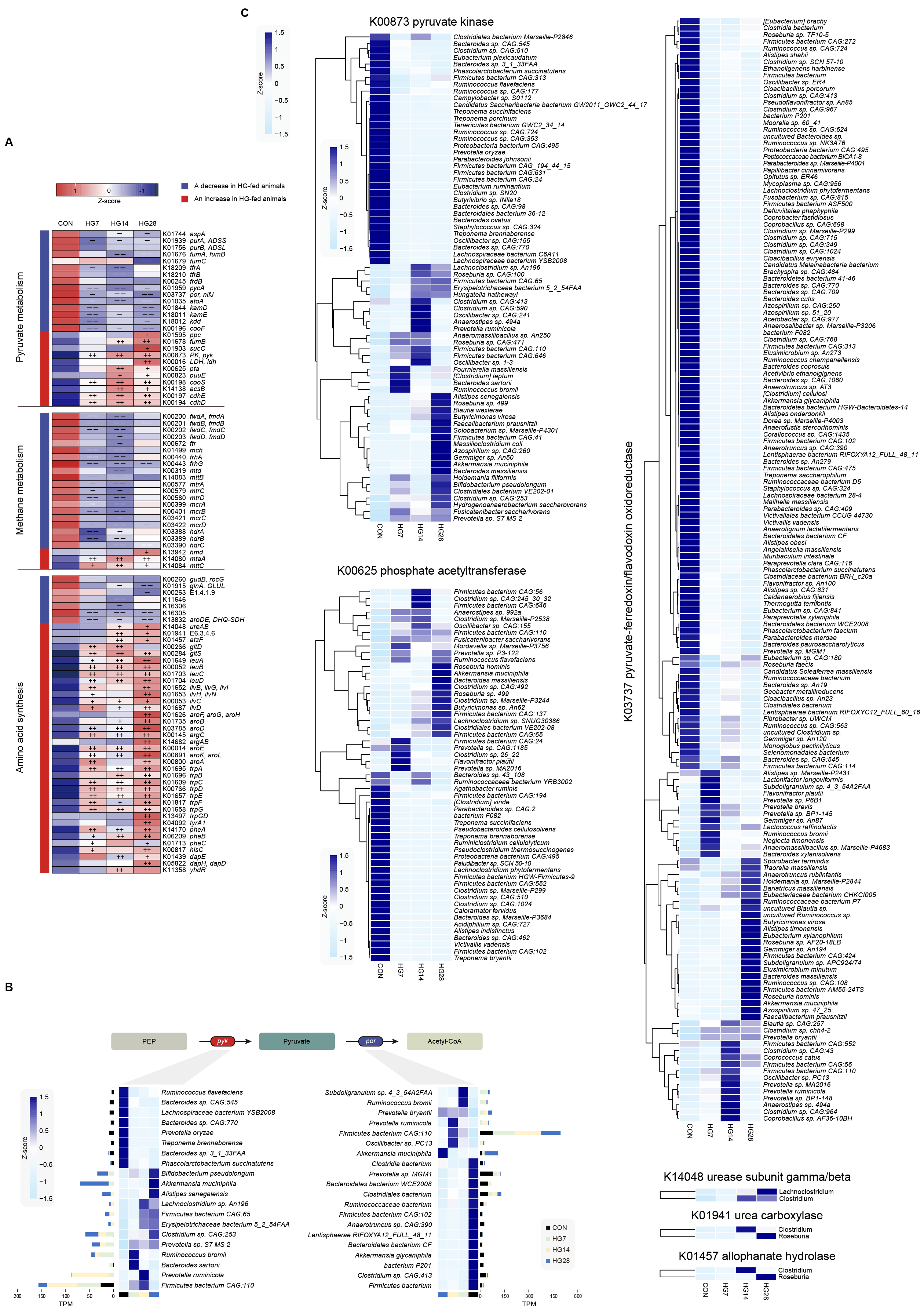

Supplement: FIG S4 [file msystems.00915-21-sf004.tif]
