# Supplementary material for: Metagenomic Sequencing Reveals that High-Grain Feeding Alters the Composition and Metabolism of Cecal Microbiota and Induces Cecal Mucosal Injury in Sheep
Source: mSystems. 2021 Oct 5;6(5):e00915-21. doi: 10.1128/mSystems.00915-21 (PMC8547435; doi:10.1128/mSystems.00915-21)
Supplement: TABLE S4 [file msystems.00915-21-st004.docx]

**Table S4. Ingredient and chemical compositions of the two diets.**

| Items |  | Result for diet: | |
| --- | --- | --- | --- |
|  |  | CON | HG |
| Ingredient composition on DM basis, % | |  |  |
|  | Oat hay | 63.40 | 26.00 |
|  | Alfalfa hay | 33.00 | 14.00 |
|  | Corn meal | 0 | 34.20 |
|  | Wheat meal | 0 | 18.00 |
|  | Soybean meal | 0 | 4.20 |
|  | CaCO_3_ | 1.00 | 1.00 |
|  | NaCl | 0.40 | 0.40 |
|  | CaHPO_4_ | 1.20 | 1.20 |
|  | Mineral and vitamin supplement^1^ | 1.00 | 1.00 |
| Nutrient composition on DM basis, % | |  |  |
|  | Crude protein | 11.18 | 11.92 |
|  | Crude fat | 2.09 | 2.49 |
|  | Crude fiber | 28.41 | 12.9 |
|  | Neutral detergent fiber | 44.45 | 24.54 |
|  | Acid detergent fiber | 19.52 | 10.15 |
|  | Crude ash | 8.34 | 4.53 |
|  | Total starch | 3.25 | 32.34 |
| Digestible energy, MJ/kg | | 8.88 | 11.73 |

^1^Contained 16% calcium carbonate, 102 g/kg of Zn, 47 g/kg of Mn, 26 g/kg of Cu, 1,140 mg/kg of I, 500 mg/k of Se, 340 mg/kg of Co, 17,167,380 IU/kg of Vitamin A, 858,370 IU/kg of vitamin D, and 23,605 IU/kg of vitamin E.
